# Resistance of Bacteria toward 475 nm Blue Light Exposure and the Possible Role of the SOS Response

**DOI:** 10.3390/life12101499

**Published:** 2022-09-26

**Authors:** Magdalena Metzger, Ara Hacobian, Lisa Karner, Leonie Krausgruber, Johannes Grillari, Peter Dungel

**Affiliations:** 1 Ludwig Boltzmann Institute for Traumatology, The Research Center in Cooperation with AUVA, 1200 Vienna, Austria; 2 Austrian Cluster for Tissue Regeneration, 1200 Vienna, Austria; 3 Institute of Molecular Biotechnology, University of Natural Resources and Life Sciences, 1190 Vienna, Austria

**Keywords:** antimicrobial blue light, resistance, SOS response, RecA, UV light

## Abstract

The increase in antibiotic resistance represents a major global challenge for our health systems and calls for alternative treatment options, such as antimicrobial light-based therapies. Blue light has shown promising results regarding the inactivation of a variety of microorganisms; however, most often, antimicrobial blue light (aBL) therapy is performed using wavelengths close to the UV range. Here we investigated whether inactivation was possible using blue light with a wavelength of 475 nm. Both Gram-positive and -negative bacterial strains were treated with blue light with fluences of 7.5–45 J/cm^2^. Interestingly, only some bacterial strains were susceptible to 475 nm blue light, which was associated with the lack of RecA, i.e., a fully functional DNA repair mechanism. We demonstrated that the insertion of the gene *recA* reduced the susceptibility of otherwise responsive bacterial strains, indicating a protective mechanism conveyed by the bacterial SOS response. However, mitigating this pathway via three known RecA inhibiting molecules (ZnAc, curcumin, and Fe(III)-PcTs) did not result in an increase in bactericidal action. Nonetheless, creating synergistic effects by combining a multitarget therapy, such as aBL, with an RecA targeting treatment could be a promising strategy to overcome the dilemma of antibiotic resistance in the future.

## 1. Introduction

According to the ‘Antibiotic Resistance Threats Report 2019’ of the Centers for Disease Control and Prevention (CDC), more than 35 000 deaths per year are attributed to infectious diseases with antibiotic resistant microbes in the US alone [1]. There is a general agreement among health care professionals and scientists that antibiotics need to be used less frequently and more cautiously. Hence, new treatment options are needed and actively searched for. The germicidal properties of ultraviolet (UV) light (both UV-C (100–280 nm) and UV-B (280–315 nm)) are well documented. They are mainly based on the absorption by nucleic acids at approximately 260 nm, which causes cellular damage, including the formation of cyclobutane pyrimidine dimers (CPDs), pyrimidine (6–4) pyrimidone photoproducts (6–4 PPs) and the deamination of thymine [2,3,4,5,6]. Thereby, UV irradiation triggers a multitude of negative side effects, such as erythema, inflammation, temporary suppression of the immune function, and photoaging, and it contributes to carcinogenesis [7,8,9,10,11,12,13,14,15]. Nevertheless, its bactericidal properties have been demonstrated multiple times and have been used for numerous applications, e.g., the treatment of acne, eczema, vitiligo and psoriasis [16,17,18].

To bypass the negative effects of UV light, energy-rich blue light comes into the focus of research, termed as antibacterial blue light (aBL) therapy. Using visible light with longer wavelengths than UV light, in the range of 380–500 nm, has already been shown to be effective in vitro as well as in vivo for infection control [19,20,21,22,23,24]. Still, the majority of published data focuses on blue light with wavelengths close to the UV spectrum. In the present study, we investigated whether it is possible to inactivate bacteria using blue light with a relative long wavelength of 475 nm. This wavelength has already been used to significantly release nitric oxide (NO) bound to heme proteins, such as cytochrome c oxidase (Dungel et al. 2008), and promote wound healing processes (Dungel et al. 2014) [25,26].

The antibacterial effects of aBL are based on the well-known mechanisms of photodynamic therapy, the generation of intracellular reactive oxygen species (ROS) by the excitation of light-activated photosensitizers, but aBL uses endogenous photosensitizers, such as porphyrins and flavins inside the cells instead of exogenously applied ones [27,28]. As the effects of aBL on mammalian cells are reported to be less detrimental than with UV light, further research is necessary [29,30].

In eukaryotes, in most cases, DNA lesions are recognized and repaired by the nucleotide excision repair system (NER), so they do not entail detrimental consequences [31]. In prokaryotes, the presence of DNA lesions activates their own DNA damage programs through the activation of the so-called SOS response by the protein RecA. Thus, inhibition of the SOS response would increase the susceptibility of bacteria against antibacterial therapies. Furthermore, besides its antimicrobial potential, a major advantage of aBL therapy is the recently emerging wound healing promoting effects.

aBL therapy with longwave blue light could combine regenerative as well as antimicrobial effects [25,26]. Thus, the aim of the present study was to investigate the antimicrobial potential of blue light of 475 nm and to evaluate the role of the bacterial SOS response, especially RecA, in the responsiveness to aBL therapy in order to create a future protocol for enhancing bactericidal effects.

## 2. Materials and Methods

### 2.1. Bacterial Strains and Chemicals

All chemicals were purchased from Sigma-Aldrich, Burlington, USA, if not stated otherwise. Bacterial strains used in this work were *Escherichia coli* K12 (Addgene, Watertown, MA, USA), *Escherichia coli* TOP10 (Invitrogen, Carlsbad, CA, USA)*, Escherichia coli* NEB 5α (New England Biolabs, Ipswich, USA) and *Staphylococcus carnosus* (kindly provided by Dr. K. Plätzer, University of Salzburg). The respective strains were chosen based on the presence of the *recA* gene in their genome. A list stating which strains carry a functional copy can be seen in Table 1.

### 2.2. Culture Conditions

The bacterial strains were cultivated in 4 mL Lysogeny broth (LB, BP1426-2, Thermo Fisher Scientific Inc., Waltham, MA, USA) under aerobic conditions at 37 °C and 250 rpm on an orbital shaker (Edmund Bühler^®^ TH15, Hechingen, Germany).

### 2.3. Light Source

LED devices emitting 475 nm blue light (REPULS Lichtmedizintechnik GmbH, Vienna, Austria) were used for all experiments. The lamps radiate with a pulse rate of 50% and an intensity of 50 mW/cm^2^ as determined with a USB2000 spectrometer (Ocean Optics, Orlando, FL, USA). The repetition frequency was 2.5 Hz, while the irradiation duration varied from 5 to 30 min, resulting in a fluency of 7.5–45 J/cm^2^. The light source was placed directly on top of the plates with the lids closed. Absorption of the polystyrol lids was negligible.

### 2.4. Blue Light Treatment

All experiments with bacteria were performed during their exponential growth phase. Overnight cultures were diluted in fresh medium and optical density was measured at a wavelength of 600 nm (OD_600_) using a spectrophotometer (HITACHI^®^ Ratio Beam Spectrophotometer U-5100, Tokyo, Japan) and again after 30–40 min of incubation at 37 °C, 200–250 rpm.

#### 2.4.1. Planktonic Assays

**Figure 1:**
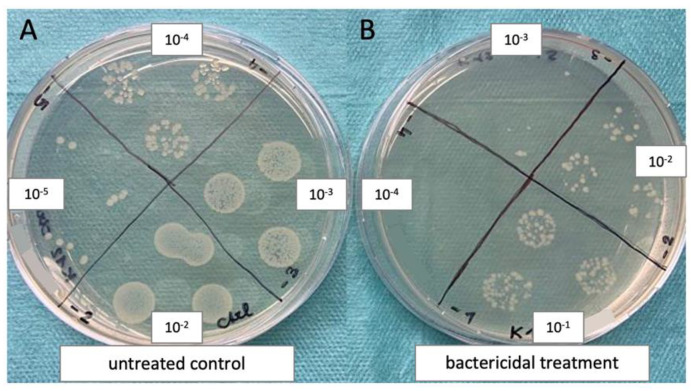
Example of the bacteria quantification method used in the present work: Three drops of 20 µL of four different bacterial dilutions were grown overnight on LB-agar plates. On the next day, distinct colonies were counted in at least two sections, and the colony forming units (CFU) were calculated. Here, *E. coli* K12 was either untreated (**A**) or treated with ciprofloxacin (**B**).

For planktonic assays, bacterial suspensions were centrifuged for 5 min at maximum speed in a table centrifuge. After discarding the supernatant, the pellet was resuspended in PBS until it was thoroughly dispersed. Next, the optical density was measured and the bacterial concentration was determined, using the formular OD_600_ = 1.0 = 8 × 10^8^ CFU/mL. The suspension was diluted in PBS to reach a final concentration of 1 × 10^7^ CFU/mL and 250 µL/well were transferred into several 24-well plates. One plate was kept in the dark for a total duration of 30 min as a control, and the remaining plates were irradiated with blue light for 5–30 min respectively, resulting in a fluence of 7.5–45 J/cm^2^. After the treatment, individual serial dilutions were prepared from each well. Next, three drops of 20 µL of four dilutions were transferred to LB-agar plates according to the method published by Miles and Misra (an example image is shown in Figure 1) [32]. Plates were incubated at 37 °C overnight, and on the next day, distinct colonies were counted and total colony forming units (CFU) were calculated.

#### 2.4.2. Assays on Agar Plates

**Figure 2:**
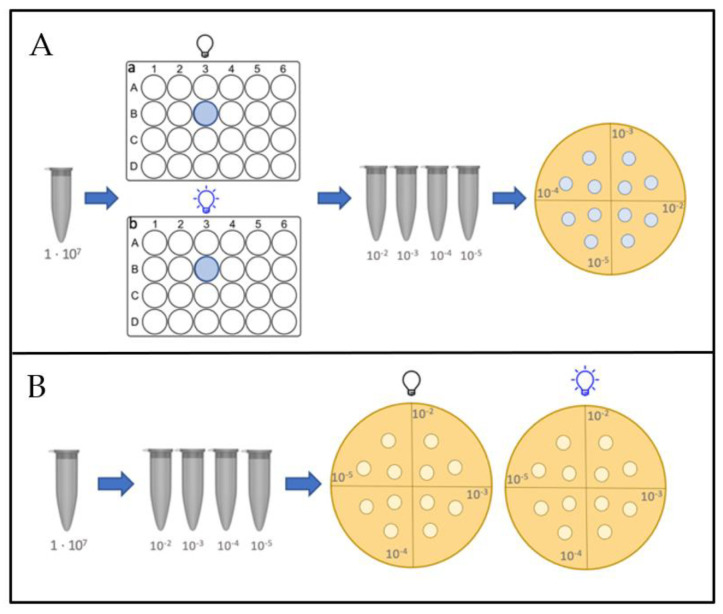
Visualization of two different workflows of blue light treatment. (**A**) planktonic assay: After preparing a bacterial suspension in PBS, aliquotes were transferred into 24-well plates and irradiated while in planctonic suspension. Next, serial dilutions were made using PBS as a dilutant, and 20 µL aliquots were transferred onto agar plates. (**B**) Agar plate assay: In order to simulate a more complex environment during treatment, bacteria were treated when growing on agar.

In order to simulate a more complex solid environment, bacteria were also grown and treated on agar plates. Overnight cultures were diluted and after 30 min, the concentration was adjusted to 1 × 10^7^ CFU/mL. A serial dilution was prepared in growth medium and 20 µL drops were transferred onto agar plates. LED lamps were placed directly on top of the petri dishes with their lids closed. Cells were further handled as described above. A schematical illustration of both protocols is shown in Figure 2.

### 2.5. Testing the Role of RecA Inhibitors for Protection against 475 nm Blue Light

Three different compounds that were already reported to inactivate one or more components of the SOS response were used to test if the efficacy of aBL therapy can be increased in the aBL-resistant bacterial strain *E. coli* K12: zinc acetate (ZnAc), Fe(III)-PcTs and curcumin (Cur) [33,34,35]. All experiments were additionally performed using *E. coli* TOP10, which lacks a functional *recA* gene (*recA^−^* control). First, minimal inhibitory concentrations were determined (Supplementary Material, Figures S4–S6), defined as the highest concentration of the respective compound that did not decrease bacterial viability. Working concentrations were 500 µM ZnAc, 300 µM Fe(III)-PcTs and 10 µM curcumin, respectively.

Planktonic bacterial cultures were incubated with the respective working concentration or PBS as control for 1 h at 37 °C on a shaker at 200–250 rpm. Next, the bacterial count was determined via OD_600_ measurements and adjusted to 1 × 10^7^ CFU/mL in LB-medium in Eppendorf tubes. Inhibitors were present in all subsequent steps. Before exposing the cells to blue light, a 1:10 serial dilution of each bacterial suspension was prepared, and three 20 µL replicates were pipetted on LB-agar plates. In addition to regular plain LB-agar plates, the experiments were also conducted on plates where the agar was supplemented with the same concentration of the respective RecA inhibiting substance to ensure long-time exposure. Afterward, the plates were irradiated with a fluence of 30 J/cm^2^ (20 min) and then incubated overnight at 37 °C.

### 2.6. Insertion of a Plasmid Carrying the Gene RecA into RecA Negative (RecA^−^) Bacterial Strains and Subsequent Blue Light Exposure

Two *recA^−^* bacterial strains (*E. coli* NEB 5α and *E. coli* TOP10) that were purchased already competent were both transformed with one of two plasmids: (1) the first one carrying the gene *recA* (termed RecA-plasmid) or (2) an identical plasmid expressing *gfp* instead (termed Ctrl-plasmid) under the control of the same *recA* promoter. The plasmid used was pMK-RQ (Invitrogen, USA). For the transformation, we followed the One Shot^®^ chemical transformation protocol by Invitrogen, Carlsbad, USA. Cells were irradiated on agar plates with 30 J/cm^2^ blue light (20 min) as described in Section 2.4.2.

### 2.7. Statistical Analysis and Calculation of Logarithmic Reduction Values

All experiments were tested for statistical significance using Ordinary One-way ANOVA with Dunnett’s multiple comparisons test by GraphPad Prism 9 (GraphPad Software, Inc.) apart from one experiment evaluating the effects of aBL on plasmid transformed bacterial strains, where a paired t-test was used. Statistical significance was accepted at *p* ≤ 0.05. Reduction of bacterial count is expressed as logarithmic reduction values (LRV), which were calculated via the following formula:

(1) $$LRV\;\left[-\right]=log10\frac{CFU_{dark}\;\left[\frac{CFU}{mL}\right]}{CFU_{treatment}\;\left[\frac{CFU}{mL}\right]}$$

## 3. Results

### 3.1. Culture Conditions during aBL Treatment Determine Therapy Outcome

**Figure 3:**
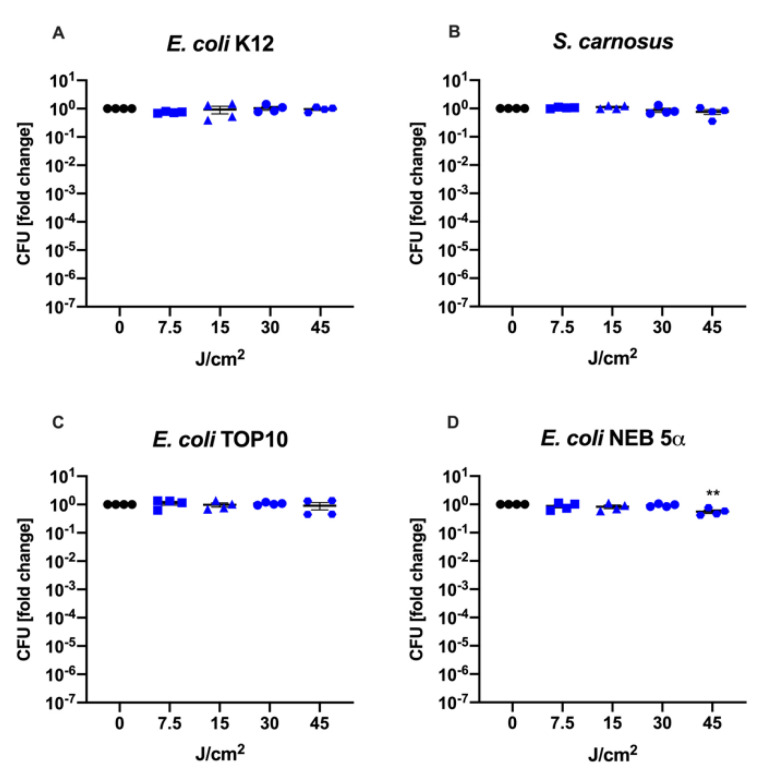
Blue light therapy conducted with four different bacterial strains in a planktonic suspension in PBS for various durations: A slight reduction of CFUs was only achieved in *E. coli* NEB 5α (**D**) LRV*_E_. _coli_* _NEB 5α_ = 0.26, *p* = 0.009) after irradiation with 45 J/cm^2^, while *E. coli* K12 (**A**), *S. carnosus* (**B**) and *E. coli* TOP10 (**C**) remained unaffected. The irradiated groups were compared to a group that was left in the dark (0 J/cm^2^). Black dots: 0 J/cm^2^, blue squares: 7.5 J/cm^2^, blue triangles: 15 J/cm^2^, blue circles: 30 J/cm^2^, blue hexagons: 45 J/cm^2^. Mean ± SEM, *n* = 4, ** *p* ≤ 0.01.

**Figure 4:**
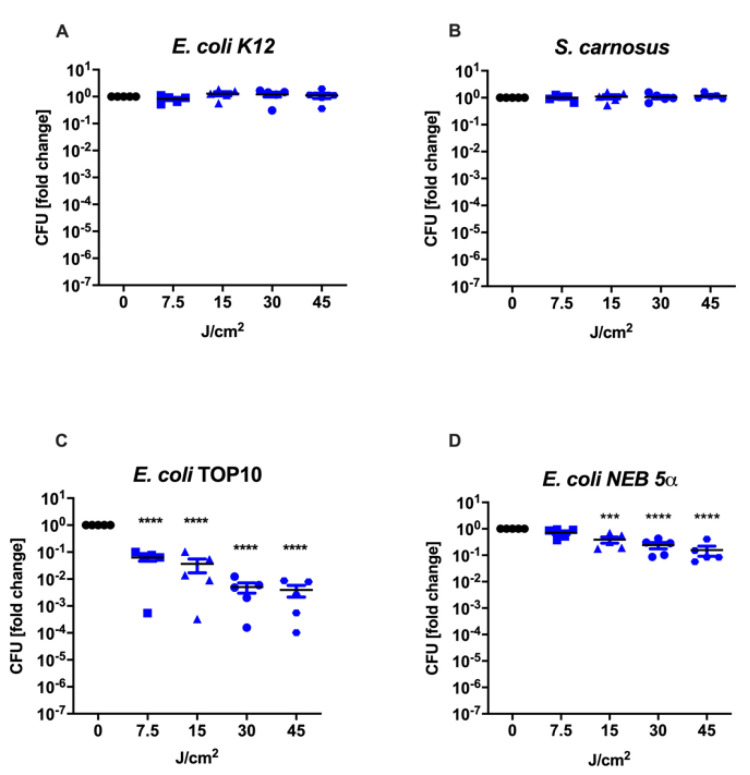
Exposure to different doses of 475 nm blue light on LB-agar: one Gram−(+) bacterial strain (*S. carnosus* (**B**)) and three Gram−(−) strains (*E. coli* K12 (**A**), *E. coli* TOP10 (**C**), *E. coli* NEB 5α (**D**)) were irradiated with either 0, 7.5, 15, 30 or 45 J/cm^2^ of blue light. While the irradiation was not able to reduce the CFU of *E. coli* K12 and *S. carnosus*, the two strains with a defective copy of the *recA* gene (*E. coli* TOP10 and *E. coli* NEB 5α) were successfully diminished. At 45 J/cm^2^, *E. coli* TOP10 was lowered by 2.40-log_10_ CFU (*p* < 0.0001) and *E. coli* NEB 5α by 0.74-log_10_ CFU (*p* < 0.0001). Black dots: 0 J/cm^2^, blue squares: 7.5 J/cm^2^, blue triangles: 15 J/cm^2^, blue circles: 30 J/cm^2^, blue hexagons: 45 J/cm^2^. Mean ± SEM, *n* = 5, *** *p* ≤ 0.001, **** *p* < 0.0001.

The effects of 475 nm blue light exposure was first tested in planktonic suspension in PBS. Notably, while *E. oli* K12, *S. carnosus* and *E. coli* TOP10 were not significantly reduced, *E. coli* NEB 5α showed a mean reduction of CFUs by 0.26-log_10_ units (*p* = 0.009) after being irradiated with a fluence of 45 J/cm^2^ (30 min). These results would suggest that overall, the used bacterial strains are not or minimally susceptible to 475 nm blue light, at least when treated in a planktonic suspension in PBS (Figure 3). However, when blue light was applied on the same four bacterial strains directly on LB-agar plates, representing a more complex environment, the results differed. While *E. coli* K12 and *S. carnosus* did not respond to increasing doses of blue light, the CFU of *recA*^−^ strains (*E. coli* TOP10 and *E. coli* NEB 5α) were significantly decreased after a dose of 7.5 J/cm^2^ (LRV*_E_. _coli_*_TOP10_ = 1.20, *p* < 0.0001) and 15 J/cm^2^ (LRV*_E_. _coli_* _NEB 5α_ = 0.36, *p* = 0.001) respectively. While *E. coli* NEB 5α was reduced by 0.74-log_10_ units after 45 J/cm^2^ (*p* < 0.0001), it resulted in a 2.40-log_10_ CFU decline in *E. coli* TOP10 (*p* < 0.0001). To conclude, solely strains without a functional copy of the gene *recA* were inactivated by 475 nm blue light when the treatment was conducted in LB-medium (Figure 4).

Since riboflavin is discussed as a potential key photosensitizer in blue light therapy, it was tested whether the presence of riboflavin in the LB-medium is the decisive factor responsible for the observed differences. However, supplying PBS with the same content of riboflavin as LB-medium (0.44 µg/mL, determined via fluorescence measurements, supplemental material, Figure S1) did not affect therapy outcome (Supplementary Material, Figure S3). Furthermore, there was no difference in riboflavin secretion among the used strains (Supplementary Material, Figure S2), suggesting that susceptibility to 475 nm blue light exposure is obtained by mechanisms other than riboflavin overproduction. Notably, a striking difference between the resistant and the susceptible strains was the presence or absence of the gene *recA*.

### 3.2. Resistance toward 475 nm Blue Light Was Not Affected by RecA Inhibitors (ZnAc, Curcumin or Fe(III)-PcTs)

**Figure 5:**
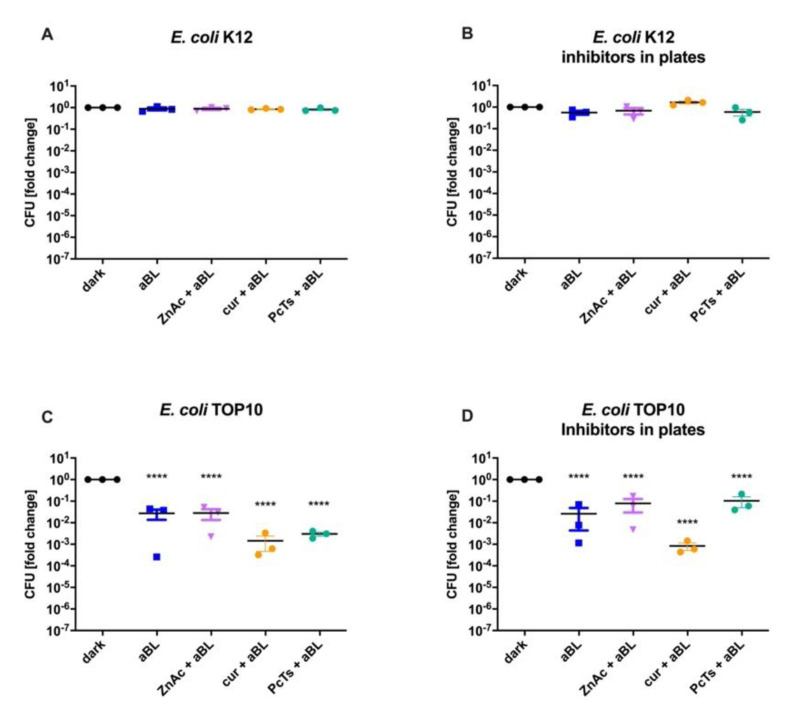
Exposure to 475 nm blue light after pretreatment with various RecA-inhibiting substances: *E. coli* K12 (**A**,**B**); contains the gene *recA* in its genome) and *E. coli* TOP10 (**C**,**D**); possesses a deletion in the gene *recA*) were irradiated with 30 J/cm^2^ blue light alone (aBL, *blue squares*) or after incubation with 500 µM ZnAc (ZnAc + aBL, *lilac triangles*), 10 µM curcumin (cur + aBL, *orange circles*) or 300 µM Fe(III)-PcTs (PcTs + aBL, *turquoise circles*). A control group was kept in the dark for the duration of the blue light treatment (dark, *black circles*). The experiment was first conducted on regular LB-agar-plates (**A**,**C**) as well as on plates containing the respective compounds (**B**,**D**). None of the compounds reduced the resistance of *E. coli* K12 toward aBL or increased the efficacy in *E. coli* TOP10. Mean ± SEM, *n* = 3, **** *p* < 0.0001.

First, the minimal inhibitory concentration (MIC) of the inhibitors was determined (Supplementary Material, Figures S4–S6). Pretreating bacteria with either ZnAc, curcumin or Fe(III)-PcTs did not reduce the resistance of the *recA*^+^ strain *E. coli* K12, neither on plain agar plates, nor when the same concentration of the named compounds was also present in the agar plates to ensure long-term exposure (Figure 5). Likewise, the antimicrobial effects of the treatment were not enhanced in the *recA*^−^ strain *E. coli* TOP10. These results suggest that RecA inactivation might have been incomplete or that other factors contribute to the resistance toward 475 nm aBL. Detailed data can be found in the Supplementary Material (Figures S7–S9).

### 3.3. Prevention of aBL Toxicity by RecA Overexpression

**Figure 6:**
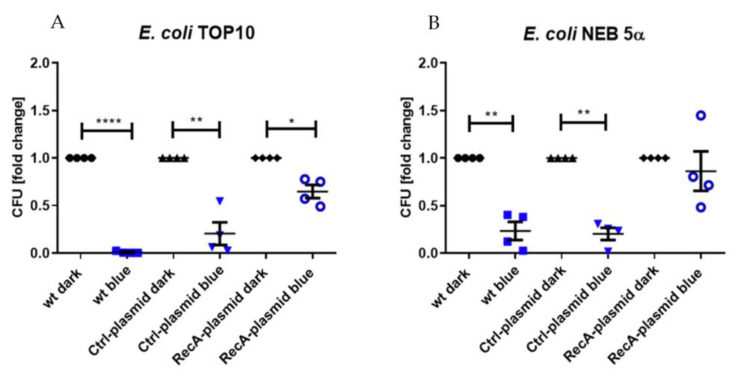
The effects of exposure to 475 nm blue light were diminished by the insertion of a plasmid carrying *recA* in both (**A**) *E. coli* TOP10 and (**B**) *E. coli* NEB 5α by 64 % and 63 %, respectively compared to the non-transformed (wt) group. Cells were irradiated with 30 J/cm^2^. Ctrl-plasmid indicates groups transformed with an inactive control plasmid. Wild-type dark: black circles, wild-type blue: blue squares, Ctrl-plasmid dark: black triangles, Ctrl-plasmid blue: blue triangles, RecA-plasmid dark: black rhombuses, RecA-plasmid blue: blue hollow circles. Mean ± SEM, *n* = 4, * *p* ≤ 0.05, ** *p* ≤ 0.01, **** *p* < 0.0001.

In two *recA^−^* bacterial strains, normally susceptible to 475 nm blue light treatment, transformation with a plasmid carrying the *recA* gene (RecA-plasmid) showed a protective effect (Figure 6). Wild-type *E. coli* TOP10, which normally shows a high susceptibility to 475 nm aBL (reduction of mean CFU by 99.18% compared to non-treated group, *p* < 0.0001), after transformation showed a reduction by only 35.21% (*p* = 0.0148). Transformation with the Ctrl-plasmid resulted in an average decrease of 79.48% (*p* = 0.0068).

Similar data were demonstrated with *E. coli* NEB 5α. aBL irradiation decreased CFUs of the non-transformed, and the Ctrl-plasmid transformed groups by 76.61 % (*p* = 0.0039) and 79.64 % (*p* = 0.0011), respectively. By transforming the strain with the RecA-plasmid, a reduction of only 13.68 % (non-significant) of CFU could be observed, suggesting that the plasmid expressing *recA* gene provided protection toward aBL.

## 4. Discussion

During the course of the 20th century, the mortality of certain diseases that were previously considered untreatable, such as syphilis, typhoid fever or plague, was considerably decreased, thanks to the discovery of antibiotics. However, due to the readily available access to these drugs, incorrect use and overuse, we are now faced with the pressing issue of increasing bacterial resistance against antibiotics and a lack of alternative treatment options for a variety of life-threating diseases.

One promising approach could be antimicrobial blue light (aBL) therapy. While the underlying mechanism of bacterial inactivation is still the subject of current research, evidence indicates that light with a wavelength in the blue range (>380 nm) can activate endogenous photosensitizing molecules within the cells (such as flavins and porphyrins) to produce cell-destructing ROS [27,36].

One major advantage of light-based therapies in contrast to antibiotic drugs is a presumably lower probability of the development of resistance due to the in situ multitarget mechanism of action. There are little data about bacterial resistance toward blue light published to date. Guffey et al. reported a decline in the kill rate after four consecutive irradiation cycles with 405 nm on *S. aureus.* Interestingly, before the decrease in efficacy, the treatment became more efficient with each cycle. Still, the lower bactericidal effects after cycle 4 were statistically significant [37]. Rapacka-Zdonczyk et al. demonstrated that sub-lethal aBL therapy with 411 nm blue light increased treatment tolerance after four consecutive cycles of exposure to aBL and subcultivating. Furthermore, their findings suggest a SOS-response depended, specifically *umuC*-dependent effect of tolerance acquisition [38]. Similar findings were reported using UV irradiation (254 nm), where a reduction in treatment success was caused by mutations in genes involved in DNA repair and replication [39].

In contrast to previous studies, blue light of longer wavelengths has been shown to trigger positive regenerative effects. Light of 470 nm increased angiogenesis and reduced necrotic damage in an ischemia-disturbed wound healing model in rats [26]. It also enhanced wound healing in an excision wound model [40]. Other groups reported positive effects in the healing of chronic wounds [26,41]. Depending on light parameters, blue light also reduced proliferation of skin cells, aiding in the treatment of hypertrophic scars [42,43]. Thus, we were interested in using blue light with a wavelength of 475 nm and an intensity of 50 mW/cm^2^ on Gram-positive as well as -negative bacterial strains to test the antimicrobial potential. While two strains were successfully reduced after being irradiated with as little as 7.5 J/cm^2^ (*E. coli* TOP10, LRV = 1.20, *p* < 0.0001) or 15 J/cm^2^ (*E. coli* NEB 5α, LRV = 0.36, *p* = 0.0001), the other two strains remained unresponsive (Figure 4). Interestingly, efficacy depended not only on the bacterial strain but also the environment that the treatment was performed in. When the bacterial suspensions were irradiated in planktonic suspension in PBS, a standard method for photodynamic assays, there was no or a minimal decline in colony forming units (CFU; Figure 3), while the same strains were susceptible to the treatment when treated directly on LB-agar plates (Figure 4). One possible reason for this could be that the two susceptible strains produced excessive amounts of riboflavin, which is the precursor of flavin mononucleotide (FMN) and flavin adenine dinucleotide (FAD) and believed to be one of the major photosensitizing compounds within the cells [27]. However, when measuring the riboflavin content of the cultivation medium after overnight incubation, no significant differences were detected among the different bacterial strains (Supplementary Material, Figure S2). Furthermore, when supplying PBS with the same amount of riboflavin that can be found in LB-medium, the therapy did not result in any reduction of an otherwise receptive strain in regular LB-medium (supplementary material, Figure S3). These results suggest that extracellular riboflavin did not play an integral role in the efficacy of aBL therapy. Besides flavin-based photosensitizers, recently it was also shown that bacteria produce different amounts and types of porphyrins depending on the composition of their growth medium [44]. For example, glutamate in the culture medium can be converted to 5-aminolevulinic acid by bacteria, the precursor of all porphyrins [45]. Therefore, there is also the possibility that the LB-medium contains vital molecules needed for the production of the responsible internal photosensitizing molecules. This is especially important considering the vast variety of host enzymes, proteins and antioxidants present in a clinical setting treating patients. Further experiments are required to identify the reasons for the dissimilarities in the outcomes between the treatment in a planktonic PBS suspension versus on solid agar surface.

However, comparing the genotypes of the strains revealed that those strains that were susceptible to 475 nm blue light (*E. coli* TOP10 and *E. coli* NEB 5α) carried a non-functional copy of the gene *recA*. This gene encodes the key molecule involved in the bacterial SOS response called RecA, which facilitates DNA repair and homologous recombination [46,47]. The protein RecA becomes activated when the replication fork stalls due to DNA damage and stimulates the repressor molecule LexA to cleave itself in order to allow translation of downstream genes involved in the SOS response [48,49,50]. These genes include *uvrAB* (involved in NER), *sulA* (inhibits cell division), *umuCD* (error-prone DNA polymerase V) and many more [51]. Additionally, the expression of the genes *recA* and *lexA* themselves are induced in the early phases of the SOS response [52].

It is well known that antibiotics, which act by inhibiting DNA replication, as well as UV rays, ROS and bacteriophages are able to induce this RecA-regulated repair program in bacteria [53,54,55,56,57]. Furthermore, Rapacka-Zdonczyk et al. reported that also aBL therapy with a wavelength of 411 nm and a fluence of 150 J/cm^2^ triggered the SOS response, most likely due to its ROS-dependent mechanism of action [38].

Preventing the activation of this pathway deprives the cells of DNA repair mechanisms and thereby increases their vulnerability toward bactericidal treatments. Besides that, the SOS response also contributes to resistance development by engaging the error-prone DNA polymerase V as well as by promoting the horizontal gene transfer of plasmids that often contain virulence factors and antibiotic resistance genes [58,59]. Hence, these properties make the targeted modulation of the bacterial SOS response a valid and therapeutically attractive approach for antibacterial treatments.

To investigate the importance of RecA in the bacterial inactivation by blue light, three chemical compounds that were previously reported to inhibit the mentioned protein were tested in their ability to reverse the resistance of *E. coli* K12.

First, zinc acetate (ZnAc) has been shown to inhibit ciprofloxacin- as well as zidovudine-induced *recA* gene expression at 200 µM in a Shiga toxin-producing *E. coli* strain [33]. Sterer et al. found that adding 1 % (*w*/*v*) of ZnAc when irradiating a biofilm with a light source emitting a wavelength of 400–500 nm and a dose of 41, 82 and 164 J/cm^2^ increased the inactivation of malodor-producing bacteria compared to aBL treatment alone [60]. In the present work, 475 nm aBL therapy was performed with a concentration of 500 µM ZnAc with *E. coli* K12 and *E. coli* TOP10. After incubating both strains with and without ZnAc for one hour, the bacterial suspensions were irradiated for 20 min, resulting in a fluence of 30 J/cm^2^. While the CFU of *E. coli* TOP10 were reduced, the *recA*^+^ strain *E. coli* K12 was not influenced by ZnAc, and no reduction in CFU was observed.

Second, the phenolic compound curcumin was found by Oda et al. to inhibit UV-induced, SOS-regulated *umuC* (polymerase V) expression in a dose-dependent manner at a 50 % inhibition value of 7.8 µg/mL in *Salmonella typhimurium* [61]. Moreover, Bellio et al. also saw a diminished SOS response using 4 µg/mL curcumin after inducing it with levofloxacin [35]. Similar to ZnAc, 10 µM curcumin (or 3.7 µg/mL) did also not attenuate the resistance of *E. coli* K12 toward 475 nm blue light. The reduction of CFUs in *E. coli* TOP10 was consistent, regardless of the pretreatment with curcumin: LRV*_E_*. *_coli_* _TOP10 aBL_ = 3.10 (*p* < 0.0001), LRV*_E_*. *_coli_* _TOP10 aBL + cur_ = 2.95 (plain LB-agar plates, *p* < 0.0001) and LRV*_E_*. *_coli_* _TOP10 aBL + cur_ = 3.08 (supplemented LB-agar plates, *p* < 0.0001).

Third, the anionic, aromatic molecule Fe(III)-phthalocyanine tetrasulfonate (Fe(III)-PcTs) which is able to bind to the cationic DNA was used. Fe(III)-PcTs was shown to successfully inhibit the SOS response and thereby potentiated the bactericidal effect of ciprofloxacin. It exerted its action through not only blocking the ATPase, DNA binding and strand exchange activities, but also by stopping the autoproteolysis of LexA [34]. Here, a pretreatment with 300 µM Fe(III)-PcTs for one hour before being irradiated with 30 J/cm^2^ aBL did not reduce the resistance of *E. coli* K12 toward 475 nm blue light as well.

In summary, interestingly, none of the tested inhibitors showed therapy-enhancing effects, at least under the chosen experimental conditions (Figure 5). We carefully correlated the concentrations with those reported in literature and additionally checked the minimal inhibitory concentrations of every reagent (supplemental material, Figures S4–S6). It is not unusual for drugs targeting a multifunctional protein to require a higher dose in living cells compared to isolated proteins. Nautiyal et al. found a considerable difference between the half maximal inhibitory concentration (IC_50_) of the RecA inhibitor suramin in regard to isolated RecA protein compared to live bacterial cells [62]. Therefore, we also tested higher concentrations wherever possible. However, the approach via inhibitors had limits, as too high concentrations affected the viability of the cells.

Nonetheless, as the lack of functional *recA* gene was a striking feature of susceptible strains, we tested another approach to investigate its role by inserting a plasmid carrying the gene. Indeed, transforming *recA*^−^ strains *E. coli* TOP10 and *E. coli* NEB 5α with a *recA* containing plasmid protected both strains from the bactericidal effects of 475 nm blue light. Faster growing bacterial cultures in general tend to be more susceptible toward antibacterial drug treatments [63,64,65]. Therefore, a control group was transformed with a plasmid containing *gfp* instead of *recA,* which excluded effects of altered replication times (supplementary material, Figure S10). These data suggest the importance of *recA* in the tolerance toward 475 nm blue light and that it could be a worthwhile target for future therapeutic strategies.

## 5. Conclusions

In conclusion, aBL at 475 nm had a selective effect on the inhibition of bacterial strains. There was a striking difference of efficacy depending on the experimental conditions as well as the genotype of the strains. The data suggest that the SOS response-inducing protein RecA plays an integral role in resistance against 475 nm blue light. The modulation of the bacterial SOS response would not only benefit aBL therapy, but also antibacterial drug treatments, which are still the standard protocol for the care of infections, making it a promising strategy to overcome the dilemma of antibiotic resistance.

## Figures and Tables

**Table 1:** Bacterial strains used in this work and the presence of the gene recA.

Bacterial Strain	Functional Copy of the Gene *recA*
*E. coli* K12	+
*E. coli* TOP10	−
*E. coli* NEB 5α	−
*S. carnosus*	+

## Data Availability

Data supporting reported results are available upon request.

## Supplementary Materials

The following supporting information can be downloaded at: https://www.mdpi.com/article/10.3390/life12101499/s1, Figure S1: Standard regression of riboflavin, Figure S2: Fluorescence measurements to compare the riboflavin content in the supernatant of different bacterial strains, Figure S3: Antibacterial blue light therapy conducted in PBS which was supplemented with 0.44 µg/ml riboflavin, Figure S4: Growth curve of E. coli K12 incubated with various concentrations of ZnAc measured in a clear 24-well plate on a plate reader, Figure S5: Incubating E. coli K12 with Fe(III)-PcTs with concentrations up to 10 mM did not result in reduction of CFUs, Figure S6: Exposing E. coli K12 to increasing concentrations of curcumin, Figure S7: Antimicrobial blue light therapy in LB-medium with or without ZnAc pretreatment, Figure S8: Pretreatment with 10 µM curcumin before aBL therapy with a fluency of 30 J/cm2 did not cause any deviations from the usual response to the therapy in E. coli K12 and E. coli TOP10, Figure S9: Bacterial inactivation by aBL therapy combined with 300 µM Fe(III)-PcTs in an effort to inhibit RecA, Figure S10: Growth curve of transformed bacterial strains.
