# Supplementary material for: Resistance of Bacteria toward 475 nm Blue Light Exposure and the Possible Role of the SOS Response
Source: Life (Basel). 2022 Sep 26;12(10):1499. doi: 10.3390/life12101499 (PMC9605056; doi:10.3390/life12101499)
Supplement: Supplementary file 1 [file life-12-01499-s001.zip › life-1918737-supplementary.pdf]

Supplementary material

1. Determining riboflavin content in LB-medium

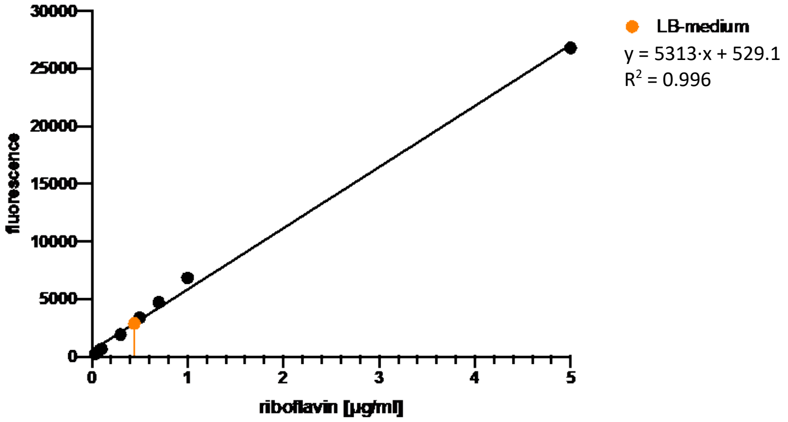

Figure S1. Standard regression of riboflavin: The fluorescence at 465 ± 35 nm excitation and 535 ± 25 nm emission of LB-medium was measured and interpolated into a riboflavin standard regression. It was determined that the fluorescence of the medium corresponded to 0.44 µg/ml riboflavin.

2. There was no overproduction of riboflavin detected among the aBL therapy susceptible bacterial strains

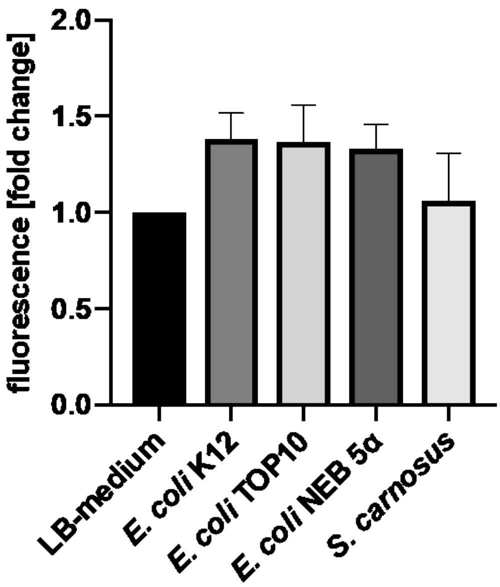

Figure S2. Fluorescence measurements to compare the riboflavin content in the supernatant of different bacterial strains: Three *E. coli* strains and one *Staphylococcus* strain were grown in LB-medium over night and the fluorescence of the cell-free supernatant was determined on the next day. Values were referred to plain LB-medium and compared to each other. Strains susceptible to aBL did not show increased fluorescence. Mean ± SEM, n = 3

3. Supplementing PBS with riboflavin has no effect on aBL therapy outcome

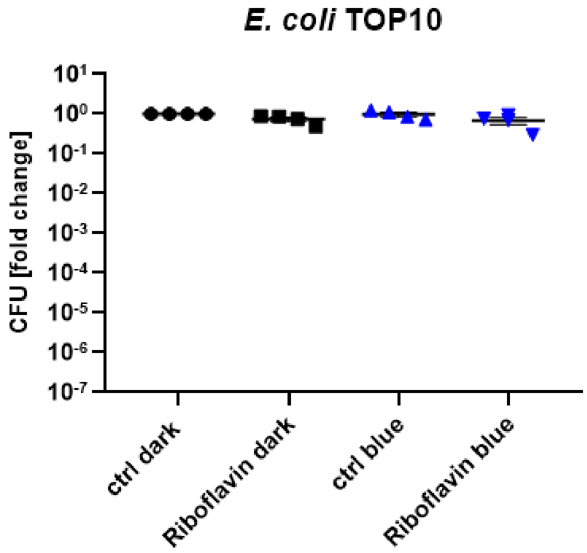

Figure S3. Antibacterial blue light therapy conducted in PBS which was supplemented with 0.44 µg/ml riboflavin: While *E. coli* TOP10 is usually inactivated by blue light when the therapy is performed in LB-medium, the strain was not susceptible in PBS (**ctrl blue**). Likewise, the addition of riboflavin as an effort to mimic a potential photosensitizing effect of LB-medium did not reduce CFUs, neither alone (**riboflavin dark**) or when irradiated with 30 J/cm^2 blue light (**riboflavin blue**) compared to an untreated group (**ctrl dark**). Mean ± SEM, n = 4

4. Determination of the minimal inhibitory concentrations of potential RecA inhibitor molecules

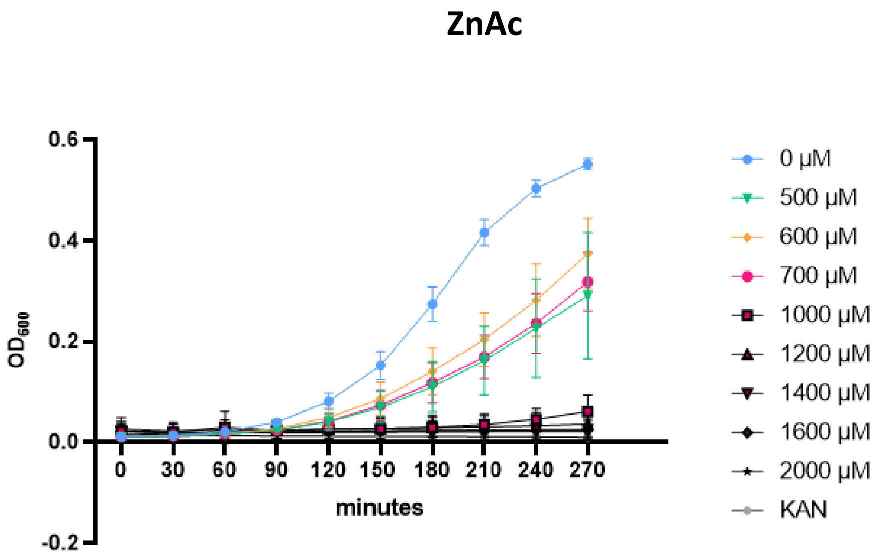

Figure S4. Growth curve of *E. coli* K12 incubated with various concentrations of ZnAc measured in a clear 24-well plate on a plate reader: While all concentrations of ZnAc reduced generation time compared to the untreated group (**0 µM**), a clear growth inhibition was observed with 1000 µM ZnAc and higher, as well as with 30 µg/ml kanamycin (**KAN**). n = 3, Mean ± SD.

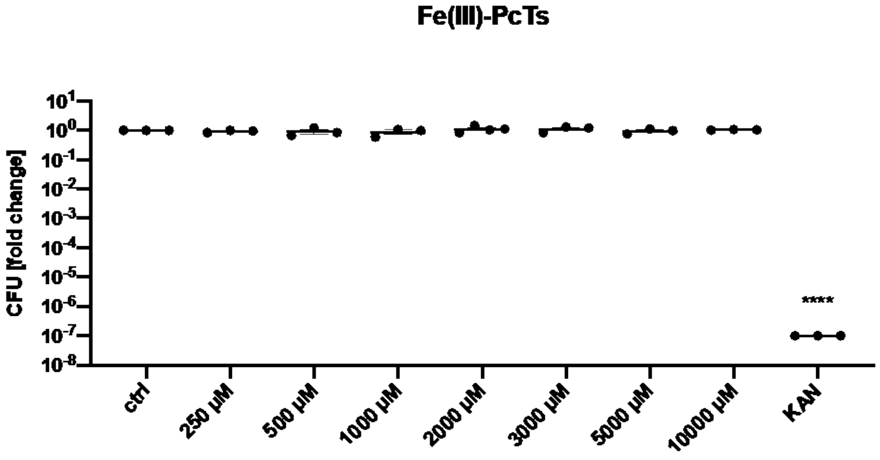

Figure S5. Incubating *E. coli* K12 with Fe(III)-PcTs with concentrations up to 10 mM did not result in reduction of CFUs. Solely a control group that received 30 µg/ml kanamycin was not able to grow (LRV_{KAN} = 7, P < 0.0001). n = 3, mean ± SEM, **** P < 0.0001.

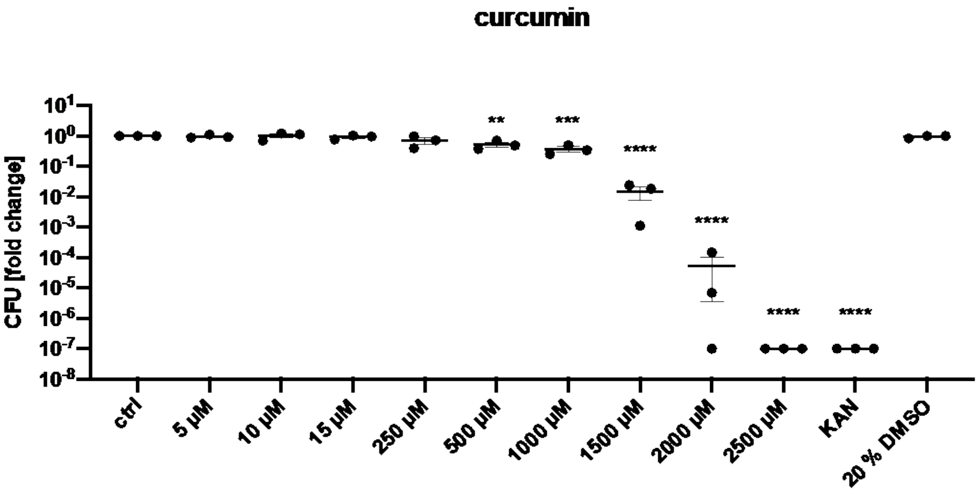

Figure S6. Exposing *E. coli* K12 to increasing concentrations of curcumin: While up to 250 µM were readily tolerated by the Gram-negative bacterial strain, 500 µM led to a 0.29-$\log_{10}$ value decrease of CFU (P = 0.033). Furthermore, $LRV_{1000\ \mu M\ cur}$ = 0.44 (P = 0.0002), $LRV_{1500\ \mu M\ cur}$ = 1.84 (P < 0.0001), $LRV_{2000\ \mu M\ cur}$ = 4.29 (P < 0.0001) and $LRV_{2500\ \mu M\ cur}$ = 7 (P < 0.0001,) just as the kanamycin group (**KAN**). Since curcumin was dissolved in DMSO, a group was included which received the same volume of DMSO as the group with the highest concentration of curcumin (2500 µM) to be able to exclude that growth inhibiting effects stem from DMSO alone. It is shown here that DMSO at a final concentration of 20 % did not cause any decrease in bacterial viability. All groups were compared to an untreated control group (ctrl). n = 3, mean ± SEM, ** P ≤ 0.01, *** P ≤ 0.001, **** P < 0.0001.

5. Blue light therapy in combination with RecA inactivating molecules

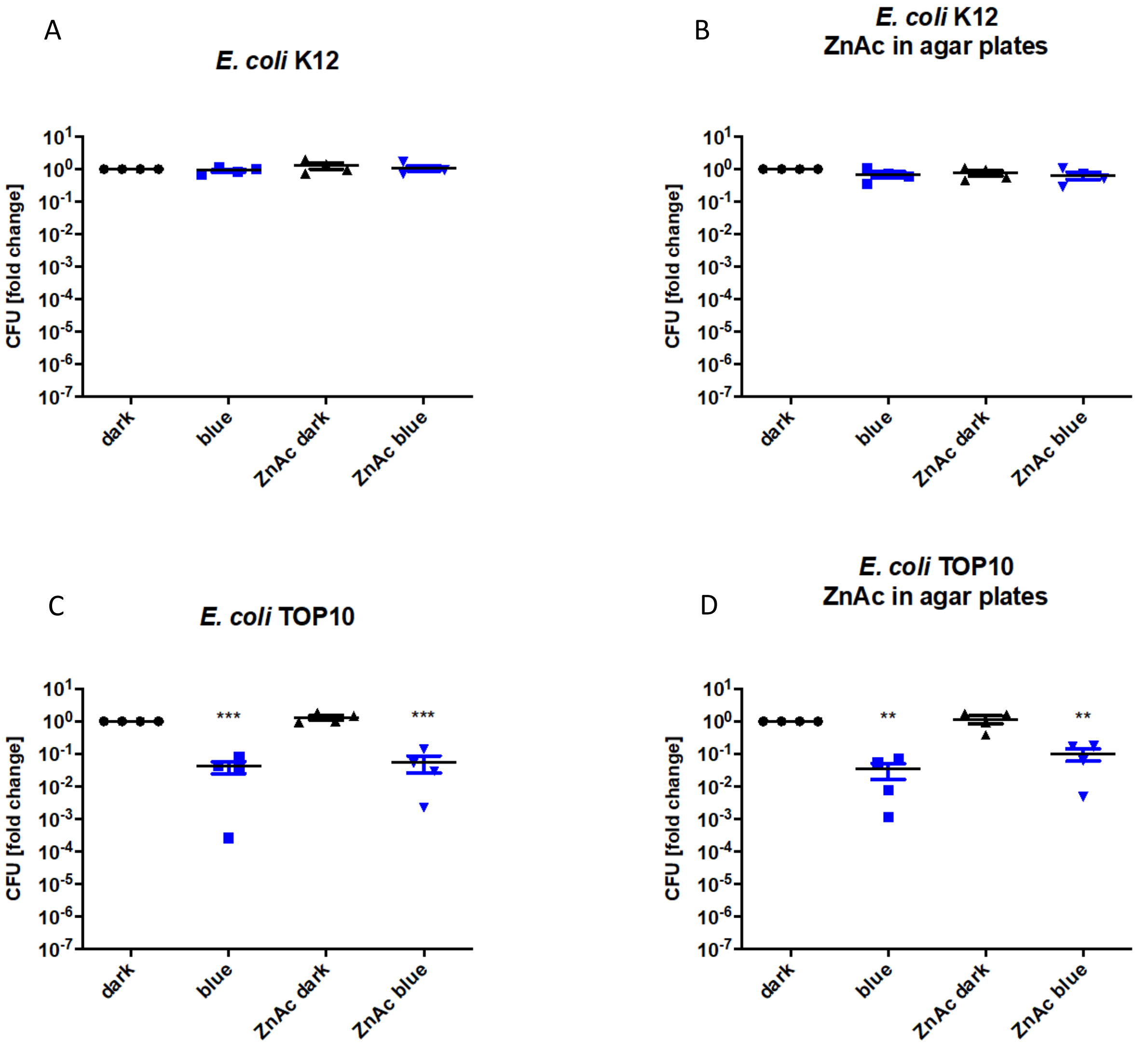

Figure S7. Antimicrobial blue light therapy in LB-medium with or without ZnAc pretreatment: *E. coli* K12 (**A, B**; contains the gene *recA* in its genome) and *E. coli* TOP10 (**C, D**; possesses a deletion in the gene *recA*) were irradiated with 30 J/cm^2 blue light alone (**blue**) or after incubation with 500 µM ZnAc (**ZnAc blue**). A control group was kept in the dark for the duration of the aBL therapy (without ZnAc: **dark**; with ZnAc: **ZnAc dark**). The experiment was first conducted in regular LB-agar-plates (**C**) as well as in plates containing 500 µM ZnAc (**B, D**). While *E. coli* K12 did not respond to aBL at all, the CFU of *E. coli* TOP10 were significantly lowered in the regular therapy group as well as with ZnAc. In plain agar plates: $LRV_{E.\ coli\ \text{TOP10 blue}}$ = 1.38 (P = 0.0002), $LRV_{E.\ coli\ \text{TOP10 ZnAc blue}}$ = 1.25 (P = 0.0002). In ZnAc-containing plates: $LRV_{E.\ coli\ \text{TOP10 blue}}$ = 1.47 (P = 0.0047), $LRV_{E.\ coli\ \text{TOP10 ZnAc blue}}$ = 0.99 (P = 0.008). Groups were compared to the dark control. Mean ± SEM, n = 4, ** P ≤ 0.01, *** P ≤ 0.001.

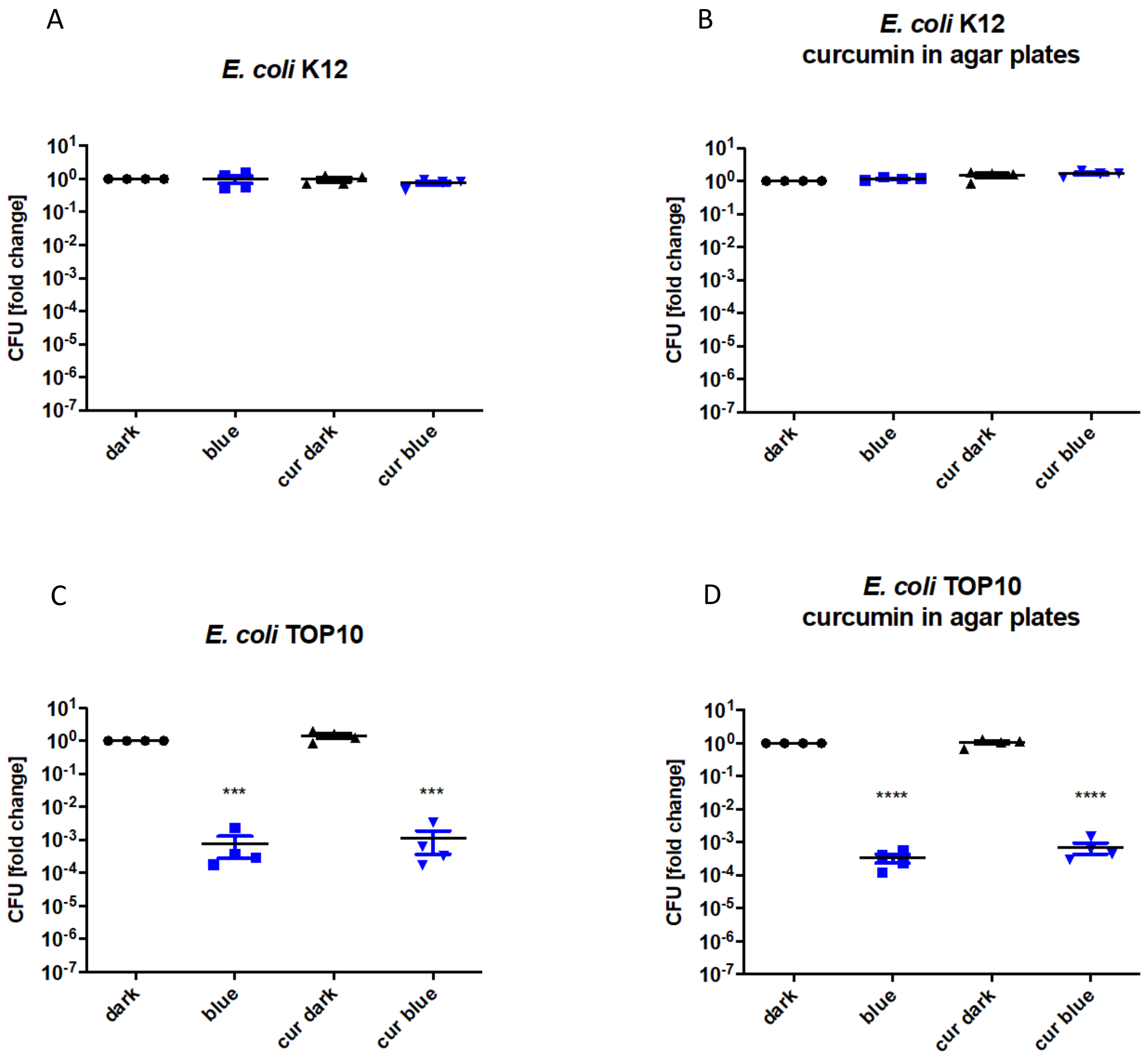

Figure S8. Pretreatment with 10 µM curcumin before aBL therapy with a fluency of 30 J/cm² did not cause any deviations from the usual response to the therapy in *E. coli* K12 and *E. coli* TOP10: The *recA*$^{-}$ strain *E. coli* TOP10 (**C and D**) was successfully inactivated by blue light without (mean $LRV_{\text{TOP10 blue}} = 3.11$, $P = 0.0004$) as well as with curcumin pretreatment (mean $LRV_{\text{TOP10 cur blue}} = 2.95$, $P = 0.0004$) compared to the non-treated control group (dark). *E. coli* K12 remained unaffected (**A and B**). The addition of curcumin to the LB-agar plates (**D**) lead to comparable decreases in *E. coli* TOP10: $LRV_{\text{TOP10 blue}} = 3.48$, $P < 0.0001$; $LRV_{\text{TOP10 cur blue}} = 3.08$, $P < 0.0001$. Mean ± SEM, n = 4, *** $P \leq 0.001$, **** $P < 0.0001$

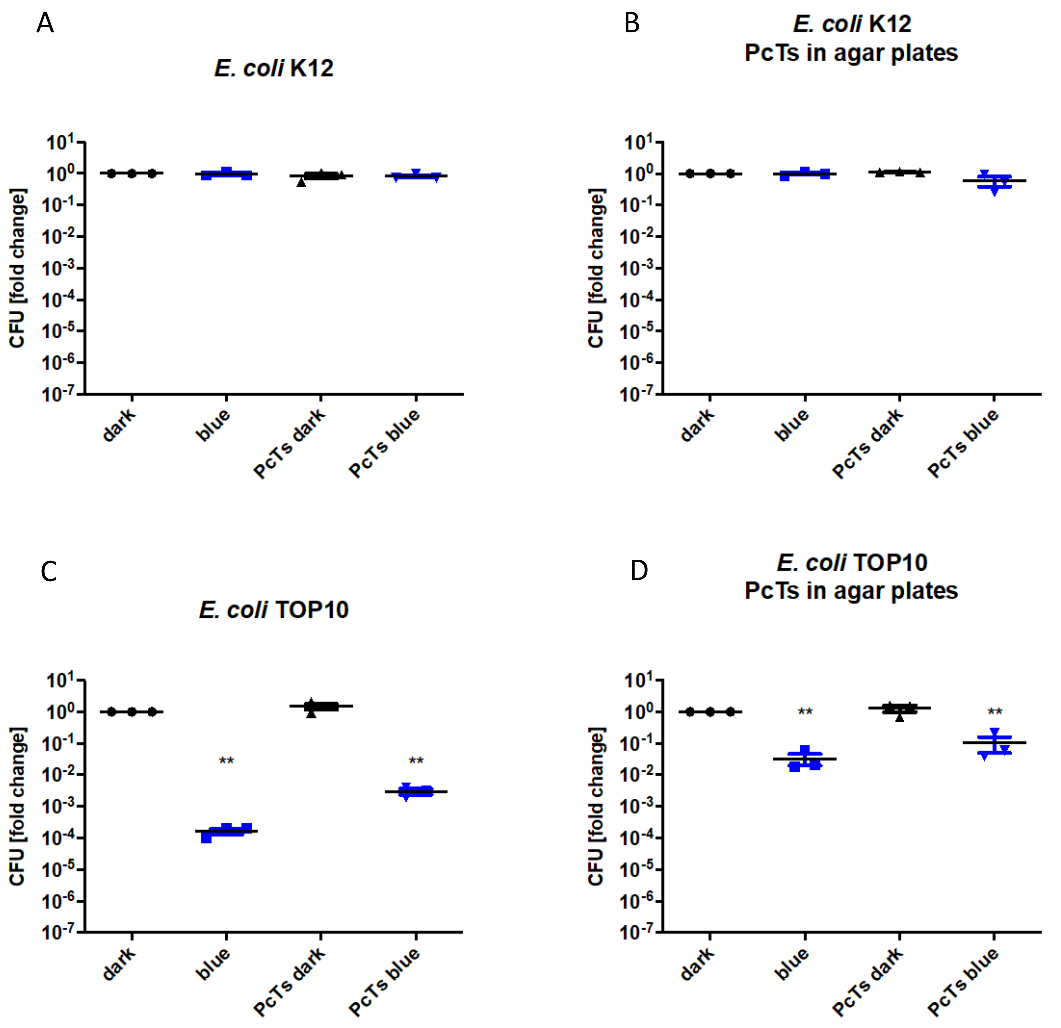

Figure S9. Bacterial inactivation by aBL therapy combined with 300 µM Fe(III)-PcTs in an effort to inhibit RecA: Bacterial cells were exposed to 30 J/cm^2 blue light with (**PcTs blue**) or without (**blue**) 300 µM Fe(III)-PcTs pretreatment. Two control groups were included: one without any treatment (**dark**) and one that received Fe(III)-PcTs as well but was kept away from the blue light (**PcTs dark**). While no change was observed in *E. coli* K12 (**A** & **B**), the *recA*$^-$ strain *E. coli* TOP10 was reduced by 3.78-$\log_{10}$ units (**C, blue**, P = 0.0042) and by 2.52-$\log_{10}$ units (**B, PcTs blue**, P = 0.0043) on plain LB-agar plates. When conducting aBL therapy on LB-agar plates containing 300 µM Fe(III)-PcTs, CFUs were less decreased in the group that was supplemented with the substance ($LRV_{E.\ coli\ \mathrm{TOP10\ blue}}$ = 1.48, P = 0.0057; $LRV_{E.\ coli\ \mathrm{TOP10\ PcTs\ blue}}$ = 0.98, P = 0.0088). Mean ± SEM, n = 3, ** Pc≤ 0.01.

6. Growth curve of transformed bacterial strains

A B

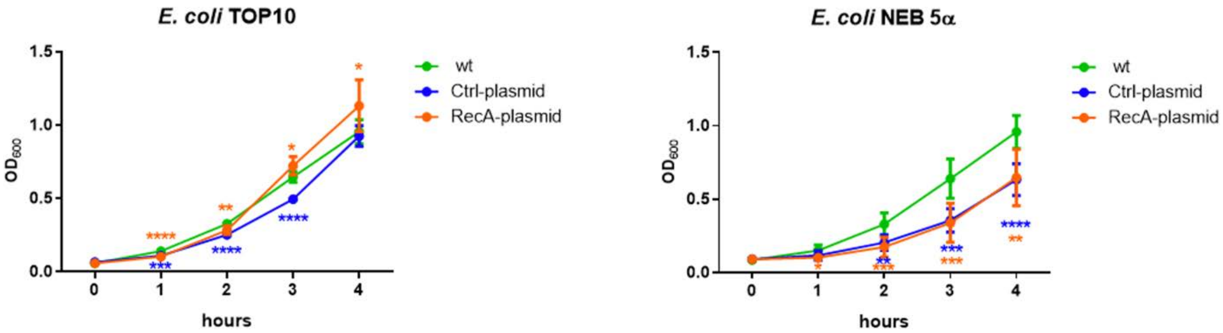

Figure S10. Growth curve of transformed bacterial strains: *E. coli* TOP10 (**A**) and *E. coli* NEB 5α (**B**) were transformed with one of two different plasmids to generate two groups for each strain: one with a plasmid containing the gene *recA* (indicated as RecA-plasmid) and one where *recA* was replaced with *gfp (*Ctrl-plasmid*)*. The increase in cell number was measured by optical density over four hours. Compared to the non-transformed cells, the growth of the Ctrl-plasmid group of *E. coli* TOP10 was significantly slower for the first three hours, while the RecA-plasmid showed increased growth after the third hour. Similarly, both plasmid-transformed groups of *E. coli* NEB 5α exhibited decreased growth in comparison to the non-transformed group. Two-way ANOVA with Dunnett's multiple comparison test. Mean ± SD, n = 3, * P ≤ 0.05, ** P ≤ 0.01, *** P ≤ 0.001, **** P < 0.0001.
